# Cognitive Sequelae of COVID‐19: Mechanistic Insights and Therapeutic Approaches

**DOI:** 10.1111/cns.70348

**Published:** 2025-03-28

**Authors:** Yu‐Hao Chen, Jing‐Shiun Jan, Chih‐Hao Yang, Ting‐Lin Yen, Tran Thanh Duy Linh, Saileela Annavajjula, Mantosh Kumar Satapathy, Shin‐Yi Tsao, Cheng‐Ying Hsieh

**Affiliations:** ^1^ Section of Neurosurgery, Department of Surgery Ditmanson Medical Foundation, Chia‐Yi Christian Hospital Chia‐Yi City Taiwan; ^2^ Chung‐Jen Junior College of Nursing, Health Sciences and Management Chia‐Yi Country Taiwan; ^3^ Department of Pharmacology School of Medicine, College of Medicine, Taipei Medical University Taipei Taiwan; ^4^ Department of Medical Research Cathay General Hospital Taipei Taiwan; ^5^ Family Medicine Training Center, University of Medicine and Pharmacy at Ho Chi Minh City Ho Chi Minh City Vietnam; ^6^ Division of Endocrinology and Metabolism, Department of Internal Medicine Taipei Taiwan

**Keywords:** brain, cognitive impairment, COVID‐19, pathophysiology, pharmacological treatment

## Abstract

**Background:**

The COVID‐19 pandemic has left an indelible mark on the world, with mounting evidence suggesting that it not only posed acute challenges to global healthcare systems but has also unveiled a complex array of long‐term consequences, particularly cognitive impairment (CI). As the persistence of post‐COVID‐19 neurological syndrome could evolve into the next public health crisis, it is imperative to gain a better understanding of the intricate pathophysiology of CI in COVID‐19 patients and viable treatment strategies.

**Methods:**

This comprehensive review explores the pathophysiology and management of cognitive impairment across the phases of COVID‐19, from acute infection to Long‐COVID, by synthesizing findings from clinical, preclinical, and mechanistic studies to identify key contributors to CI, as well as current therapeutic approaches.

**Results:**

Key mechanisms contributing to CI include persistent neuroinflammation, cerebrovascular complications, direct neuronal injury, activation of the kynurenine pathway, and psychological distress. Both pharmacological interventions, such as anti‐inflammatory therapies and agents targeting neuroinflammatory pathways, and non‐pharmacological strategies, including cognitive rehabilitation, show promise in addressing these challenges. Although much of the current evidence is derived from preclinical and animal studies, these findings provide foundational insights into potential treatment approaches.

**Conclusion:**

By synthesizing current knowledge, this review highlights the importance of addressing COVID‐19‐related cognitive impairment and offers actionable insights for mitigation and recovery as the global community continues to grapple with the pandemic's long‐term impact.

## Graphical abstract

Post‐COVID‐19 neurological syndrome, marked by cognitive dysfunction, stems from mechanisms like neuroinflammation, cerebrovascular injury, and psychological stress. This review highlights pharmacological treatments and cognitive rehabilitation as promising strategies to address cognitive impairment from acute COVID‐19 to long‐COVID phases.

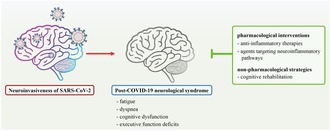

## 1 Introduction/Background

Since the emergence of coronavirus disease 2019 (COVID‐19) in 2019, the medical community has witnessed a complex and multifaceted illness. COVID‐19, primarily recognized as a respiratory syndrome, presents a wide array of symptoms affecting various organs and systems in the body. Amid the challenges in understanding the trajectory of this disease, the recovery phase of COVID‐19 patients remains shrouded in ambiguity, primarily due to the emergence of postacute complications. Beyond the initial respiratory symptoms, it has become increasingly evident that COVID‐19 has a significant neurological component. Remarkably, over 70% of COVID‐19 hospitalized patients experience neurological symptoms [1]. Recent research has shown that neurological manifestations are prevalent in 23% of cases, as reported in a comprehensive meta‐analysis [2]. Moreover, cognitive impairment (CI), which can range from 28% to 81% in incidence among COVID‐19 patients, is a growing concern [3, 4]. CI, particularly in the realm of executive functioning, has been strongly associated with reduced quality of life and heightened psychological distress in these individuals [5]. Notably, a systematic review has shed light on the alarming prevalence of attention, executive process, and memory deficits among COVID‐19 survivors [6]. Even those who have recovered and tested negative for the virus using polymerase chain reaction (PCR) testing may exhibit attention deficits [7]. Furthermore, individuals with history of cognitive and neuropsychiatric deficits seem to fare worse when infected with severe acute respiratory distress syndrome coronavirus 2 (SARS‐CoV‐2) [8]. Remarkably, literature from past respiratory illnesses (SARS‐Cov‐1 and MERS) indicate that cognitive deficits may persists for up to 5 years post‐discharge, especially among severely ill patients with acute respiratory distress syndrome (ARDS) [9]. As ongoing studies explore the persistence of postrecovery COVID‐19 symptoms, they have led to the recognition of disease entities, such as the Long COVID or Post COVID‐19 syndrome. Although we have witnessed fluctuations in the reported number of COVID‐19 cases since its onset, there remains an underlying concerns that “Post COVID‐19 neurological syndrome” (PCNS) could evolve into the next public health crisis. Although recent reviews have summarized the broad spectrum of cognitive deficits post‐COVID‐19, our review uniquely emphasizes the mechanistic insights that underlie CI and their translational relevance for targeted therapeutic strategies. By bridging current neurobiological findings with clinical implications, we aim to highlight emerging areas that warrant further research and clinical validation. Furthermore, this review critically evaluates the gaps in existing literature and offers a conceptual framework for understanding the complex interplay between persistent inflammation, neurovascular dysfunction, and neuronal injury in post‐COVID CI.

### 1.1 Clinical Presentation of COVID‐19

COVID‐19 is a disease that spares no age group, affecting individuals across the lifespan. However, its most severe cases tend to manifest in specific demographics, including newborns, men more than women, the elderly, and those with underlying systemic conditions such as coronary artery disease, diabetes mellitus, and hypertension [10]. Remarkably, up to 40% of infected individuals may remain asymptomatic, further complicating its detection and containment [11]. Among symptomatic cases, fever, shortness of breath, dry cough and fatigue are the most commonly reported symptoms. Nevertheless, COVID‐19's impact extends beyond the respiratory system, with myalgia, weakness, headache, rhinorrhea, nausea, vomiting, diarrhea, anosmia (loss of smell), and ageusia (loss of taste) affecting multiple organ systems [11].

### 1.2 Long COVID/PCNS

Understanding the phases of COVID‐19 is essential for grasping the complexities of postinfection symptoms. The UK's National Institute for Health and Care Excellence (NICE) categorizes COVID‐19 into acute, ongoing, and post‐COVID‐19 phases. Acute symptoms typically manifest within the first 4 weeks, whereas the ongoing phase spans from 4 to 12 weeks. Importantly, NICE defines post‐COVID‐19 syndrome as symptoms linked to COVID‐19 that persist for more than 12 weeks after infection and cannot be attributed to any other diagnosis [12]. Meanwhile, the World Health Organization (WHO) has recognized a clinical case definition of post COVID‐19 [13] as “symptoms occurring in individuals with a history of probable or confirmed SARS‐CoV‐2 infection, typically 3 months after the onset of COVID‐19, lasting for at least 2 months, and not explained by an alternative diagnosis”. Symptoms of post‐COVID‐19 often include fatigue, shortness of breath, cognitive dysfunction and executive function problems [13, 14]. The term “long COVID” encompasses symptoms that persist beyond the acute phase and encompasses elements of both ongoing and post‐COVID‐19 symptoms [15]. Neurological manifestations can occur in isolation or alongside other systemic symptoms, forming a significant component of long COVID. Therefore, numerous recent studies have referred to long COVID as “Post‐COVID‐19 neurological syndrome” (PCNS) PEVuZE5vdGU [16]. Epidemiological studies, along with self‐reported data, have indicated a three‐fold increase in the prevalence of neurological symptoms among long COVID sufferers. Within first 6 months following COVID infection, nearly one‐third of COVID‐19 survivors experience neurological or psychiatric disorders PEVuZE5vdGU [16]. Furthermore, SARS‐CoV‐2 may persist latently within the central nervous system (CNS) of recovered patients, potentially leading to reactivation and subsequent neurological complications [17]. It is important to note that the neurological complications during the acute phase of the disease may differ from the manifestations seen in long COVID. Encephalopathy, cerebrovascular complications, and neuroinflammatory syndromes are more common in the acute phase, whereas long COVID often presents with symptoms such as chronic fatigue and CI, including issues with memory, attention, and executive functions [15, 18]. These disparities underscore the need for a comprehensive understanding of the diverse clinical presentations of COVID‐19 and its long‐term impacts on individuals.

### 1.3 Neuroinvasiveness of SARS‐CoV‐2 and Its Enroute to CNS

SARS‐CoV2, with its single‐stranded RNA genome and a diameter of approximately 100 nm, is encapsulated in a lipid membrane housing viral genetic material [19]. The lipid plasma membrane is constructed with structural proteins like the spike glycoprotein (S) and another surface protein, hemagglutinin‐esterase. The spike glycoprotein (S) consists of three S1‐S2 heterodimers, and it plays a vital role in its binding to the angiotensin‐converting enzyme receptor (ACE2) on the host cells of infected subjects. Additionally, the RNA itself acts as a pathogen‐associated molecular pattern (PAMP), recognized by the pattern recognition receptor or toll‐like receptors (TLR) [20].

The initial stage of viral transmission commences with the binding of SARS‐CoV‐2 to the host cell through virus‐host cell interface receptors [21]. These receptors have a significant impact on the virus's tropism and pathophysiology of COVID‐19. Within the human brain, the unique receptor expressed is ACE2. Additionally, various other potential receptors, such as erythropoietin‐producing hepatocellular (Eph) receptors, ephrin ligands, transmembrane serine protease 2 (TMPRSS2), neuropilin‐1 (NRP‐1), CD147, and P2X7, have also been suggested [22]. Notably, ACE2 receptors are not limited to the olfactory epithelium; they are also found on vascular endothelial cells and smooth muscle cells in various organs, including the liver, kidney, small intestine, blood vessels, heart, and the endothelium of the brain [23].

**FIGURE 1:**
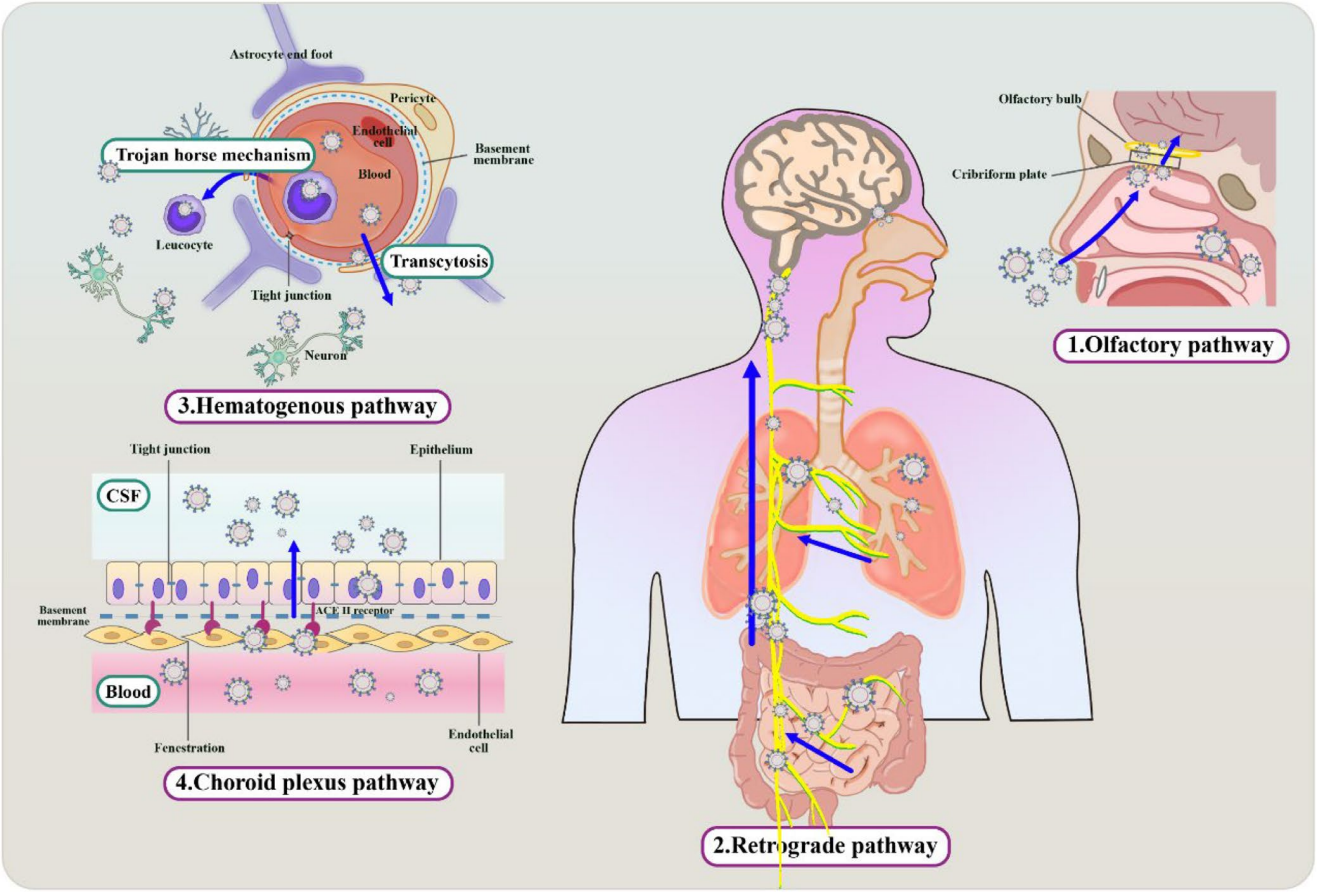
Proposed pathways for SARS‐CoV‐2 entry into the brain. A cartoon model illustrates four potential routes through which SARS‐CoV‐2 may enter the brain: (1) examining the potential for viral entry through the olfactory system, (2) exploring the possibility of retrograde transmission from peripheral nerves, (3) highlighting the potential entry through the bloodstream, and (4) demonstrating the role of the choroid plexus in viral entry.

The second stage of transmission involves the following four possible pathways (Figure 1):

1. *The olfactory pathway*: in this pathway, the S1 protein of the virus attaches to ACE2 receptors on non‐neuronal cells in the olfactory epithelium. Along with the axons of olfactory receptor cells that pass through the cribriform plate of the ethmoid bone to reach the olfactory bulb (cribriform route). From there, the virus slowly spreads to the piriform cortex and brain stem. Such interactions may explain the symptoms of loss in smell observed in COVID‐19 patients [19, 24].
2. *Retrograde transynaptic pathway*: SARS‐CoV‐2 can also enter the CNS via peripheral nerves such as the facial and vagus nerves. Retrograde transmission can occur from the gut or lung to the brain through peripheral nerves like the vagus, facilitated by the expressed NRP‐1 receptors [25]. NRP‐1 receptors are expressed in epithelial and endothelial cells of the respiratory and olfactory systems, as well as in vagal and other sensory neurons [22]. The S1 protein of the coronavirus contains a furin cleavage site, which, upon binding, activates NRP1, allowing the virus to enter the cells. This pathway of infection has been considered to associate with the commonly reported symptoms of ageusia (loss of taste) and hypogeusia (reduced taste sensitivity) [26].
3. *Hematogenous pathway*: SARS‐CoV‐2 in the general circulation may opt for this route [26](31). The blood–brain barrier (BBB), consisting of endothelial cells expressing ACE2, plays a crucial role in this pathway. The virus gains access to the CNS through the compromised tight junctions of the BBB, often due to the presence of inflammatory cytokines. It is noteworthy that considering the similarities between SARS‐CoV‐2 and the human immunodeficiency virus (HIV), a Trojan horse mechanism is another potential mode of transmission. Infected resident immune cells from the lung (leukocytes) can also act as carriers, transmitting the virus to the brain through the BBB [8, 27]. Recent research also suggested another possible entry via ACE2‐expressing CD68^+^ and CD169^+^ macrophages, which can transport the SARS‐CoV‐2 virus to other tissues [28].
4. *Choroid plexus pathway*: alternatively, SARS‐CoV‐2 can enter circulation, interact with the blood–cerebrospinal fluid barrier (BCSFB) and pass through the choroid plexus to reach the brain [8]. Viral entry through the choroid plexus has been observed in viruses such as HIV, Zika, and human polyomavirus [28, 29]. Studies on SARS‐CoV‐2 neurotropism by human brain organoids suggested that disruption of the BCSFB is conceivable [30]. However, conclusive evidence of SARS‐CoV‐2 crossing the choroid plexus and leading to neuroinvasion in humans is still lacking.

### 1.4 Pathophysiology of CI

Considering the potential long‐term adverse effects of Post‐COVID‐19 neurological syndrome on human health, it becomes crucial to gain a comprehensive understanding of the pathophysiology of CI in COVID‐19 patients and its management. The mechanisms underlying cognitive decline following COVID‐19 infection are complex and multifaceted [31]. Several interacting mechanisms have been suggested that contribute to cognitive dysfunction in PCNS.

#### 1.4.1 Direct Brain Injury

**FIGURE 2:**
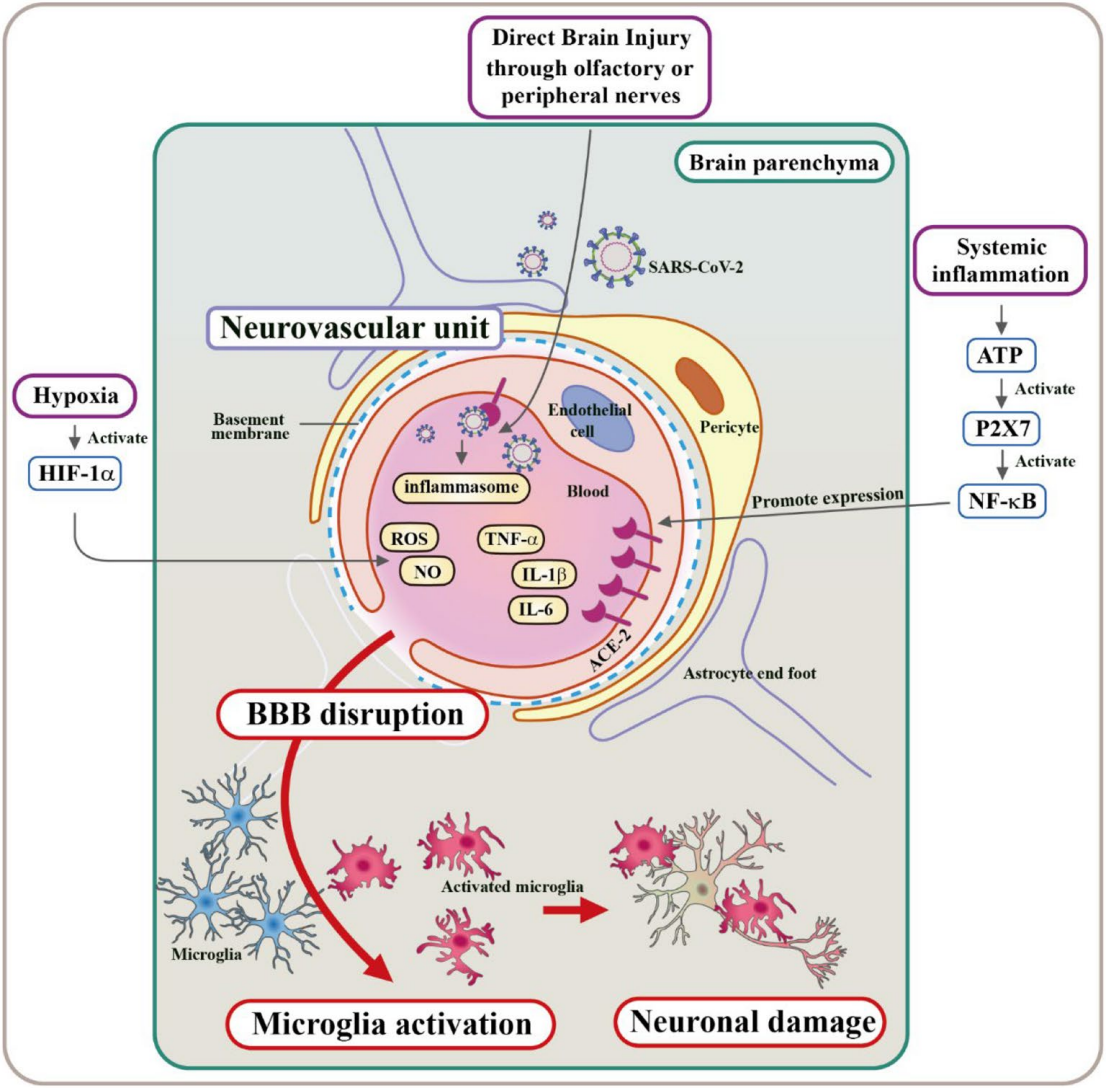
Pathophysiology of cognitive impairment in COVID‐19 patients. A cartoon figure outlines the potential pathophysiological mechanisms contributing to cognitive impairment in COVID‐19 patients, including: (1) direct viral entry induced brain injury. (2) Hypoxia induced reduction of oxygen levels may contribute to cognitive decline. (3) Highlighting the role of widespread systemic inflammation in cognitive impairment.

The response of a neurovascular unit to an exogenous trigger could result in neuroinflammation [21]. During direct viral neuroinvasion, the virus attaches to ACE2 on olfactory epithelial cells and gradually enters the CNS. Within the CNS, SARS‐CoV‐2 may activate glial cells and astrocytes, leading to the release of inflammatory mediators and increased nitric oxide (NO) production, resulting in oxidative stress and neuronal loss [32]. Additionally, the infiltration of peripheral immune cells and disrupted BBB integrity significantly contribute to neuroinflammation (Figure 2).

#### 1.4.2 Systemic Inflammation (Cytokine Storm)

Systemic inflammation is clearly observed in COVID‐19 patients. The activation of the innate immune response involves various pattern recognition receptors (PRRs) such as TLRs, P2X7 receptors, nucleotide‐binding oligomerization domain (NOD)‐like receptors, and retinoic acid‐inducible gene (RIG)‐like receptors [33]. These receptors play a pivotal role in recognizing PAMPs and damage‐associated molecular patterns (DAMPs) [34]. Extracellular ATP levels rise dramatically and may act as DAMP in a diseased state by activating P2X7 receptors and eventually creating a vicious cycle under pathological conditions [35]. This cascade begins with the upregulation of inflammatory‐related genes and the activation of the transcription complex nuclear factor kappa B (NF‐κB) [35]. The NF‐κB pathway subsequently triggers the production of proinflammatory molecules, including tumor necrosis factor‐alpha (TNF‐α), type I interferons (IFNs), interleukins (ILs) such as IL‐1, IL‐12, IL‐18, the NLR family pyrin domain containing 3 (NLRP3) inflammasome, and receptor interacting serine/threonine kinase 1 (RIPK1) [35, 36, 37]. These proinflammatory molecules not only enhance the expression of ACE2 and the epidermal growth factor receptor (EGFR) [38] but also initiate a cascade of other inflammatory cytokines and chemokines, including IL‐6, IL‐8, IL‐17, IFN, CC‐chemokine ligand 2 (CCL2), CCL3, and CXCL10, thereby exacerbating the inflammatory response [17, 36, 39, 40]. IL‐17, for instance, can induce the production of neurovascular endothelial CCL2 and CXCL1, facilitating the trans‐endothelial migration of immune cells into the CNS [21]. TNF‐α, on the other hand, has the ability to activate protein kinase‐6 and internalize VE‐cadherin, leading to the loosening of tight junctions within the BBB [36]. Consequently, these systemic inflammation‐related cytokines can breach the BBB, activate microglia, and trigger CNS inflammatory signals. Activated microglia, in turn, release IL‐1β, which has been shown to impact long‐term potentiation and memory by affecting hippocampal neurons bearing IL‐1β receptors (Figure 2) [41].

#### 1.4.3 Hypoxia

Brain hypoxia is prevalent in COVID‐19 patients with respiratory distress [25]. There is an ongoing debate regarding whether neurological signs are primary neurological symptoms or sequelae of ARDS PEVuZE5vdGU [27]. Although depression, anxiety, and post‐traumatic stress syndrome all contribute to CI in ARDS [42], accumulating evidence suggests that cognitive abnormalities can emerge independently of psychological disorders but be associated with the severity of the SARS‐CoV‐2 infection [43]. Hypoxia in the brain could stimulate the expression of hypoxia inducible factor 1 (HIF‐1α), which plays a protective role by suppressing ACE2 and TMPRSS2 expression, preventing SARS‐CoV‐2 invasion. However, HIF‐1α also contributes to the formation of the cytokine storm and the release of proinflammatory cytokines, including IL‐6. This leads to neuronal injury, the death of epithelial and endothelial cells, vascular leakage (microhemorrhages), and BBB breakdown [25], and activation of microglial cells. Activated microglia cause neuronal damage by generating cytokines and reactive oxygen species (Figure 2) [44]. Hypoxia also leads to the death of oligodendroglial cells and demyelination of white matter. In hypoxia‐activated microglia, hypercapnia can amplify the activation of the NLRP3 inflammasome, increasing the release of pro‐inflammatory IL‐1β, contributing to symptoms of CI [45].

#### 1.4.4 Dysregulated Renin–Angiotensin System (RAS)

The RAS plays a vital role in regulating renal, cardiac, and vascular physiology. ACE‐2, a key component of RAS, controls arterial blood pressure and fluid balance [19]. As the classical component of RAS, angiotensin‐2 (Ang‐II) is generated when Ang‐1 is cleaved by ACE‐1. Ang‐II binding to angiotensin II type 1 receptor (AT1R) in the vasculature causes vasoconstriction, alters vascular permeability and neurovascular coupling, and promotes neuroinflammation and oxidative stress in the CNS.

**FIGURE 3:**
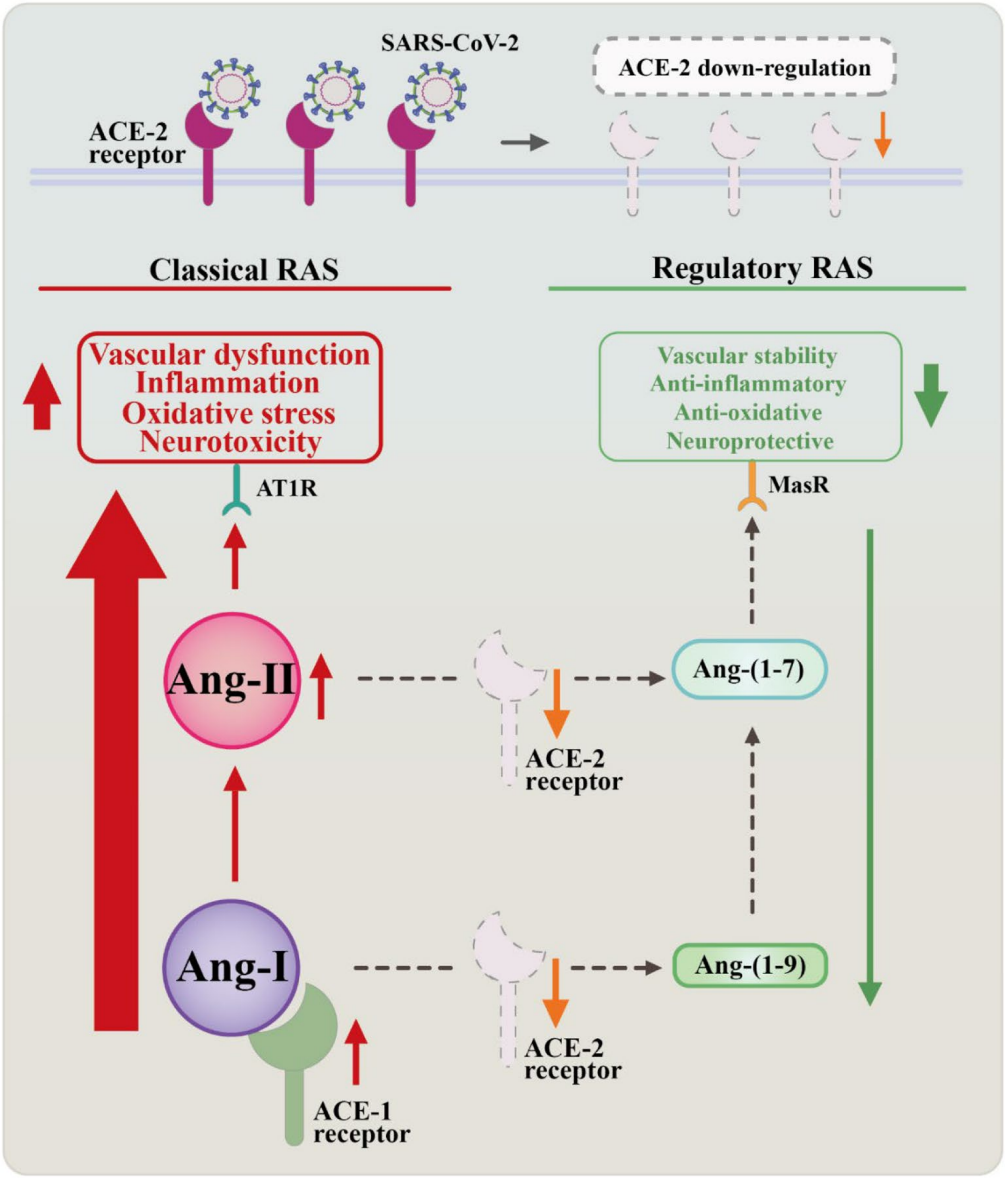
Impact of SARS‐CoV‐2 on the renin–angiotensin system (RAS). This flowchart illustrates the consequences of SARS‐CoV‐2 attachment to cells, which results in the downregulation of ACE‐2 receptor expression. This downregulation leads to the suppression of the regulatory RAS pathway and the overactivation of the classical RAS pathway.

Typically, ACE2 in the regulatory RAS counteracts the activities of the classical RAS by generating angiotensin (1–9) and angiotensin (1–7), with the degradation of Ang‐I and Ang‐II, and activation of the Mas receptor (MasR). This results in vasodilation, decreased inflammation, oxidative stress, and damage. SARS‐CoV‐2 attachment via ACE‐2 suppresses the regulatory RAS and amplifies classical RAS activity in COVID‐19 patients [46, 47]. When SARS‐CoV‐2 attacks ACE2 on vascular endothelial cells, Ang‐II, generated via ACE1, activates the Janus kinase‐signal transducer and activator of transcription (JAK–STAT) signaling pathway [38, 48]. Ang‐II acts as a proinflammatory cytokine, vasoconstrictor, and fluid retainer by increasing the secretion of aldosterone, causing oxidative stress and injury (Figure 3) [19, 49, 50, 51]. In a healthy state, ACE2 plays a role in monitoring brain function through the regulation of brain‐derived neurotrophic factor (BDNF). Reduced ACE2 activity with SARS‐CoV‐2 infection results in diminished BDNF activity, impaired maturation of neurons and microglial cells, and reduced clearance of amyloid‐beta (Aβ) peptides. This leads to memory loss, anxiety, CI, and the potential development of Alzheimer's disease (ad) [41].

#### 1.4.5 Leaky Ryanodine Receptor 2 ( **RyR2** ) Channels

**FIGURE 4:**
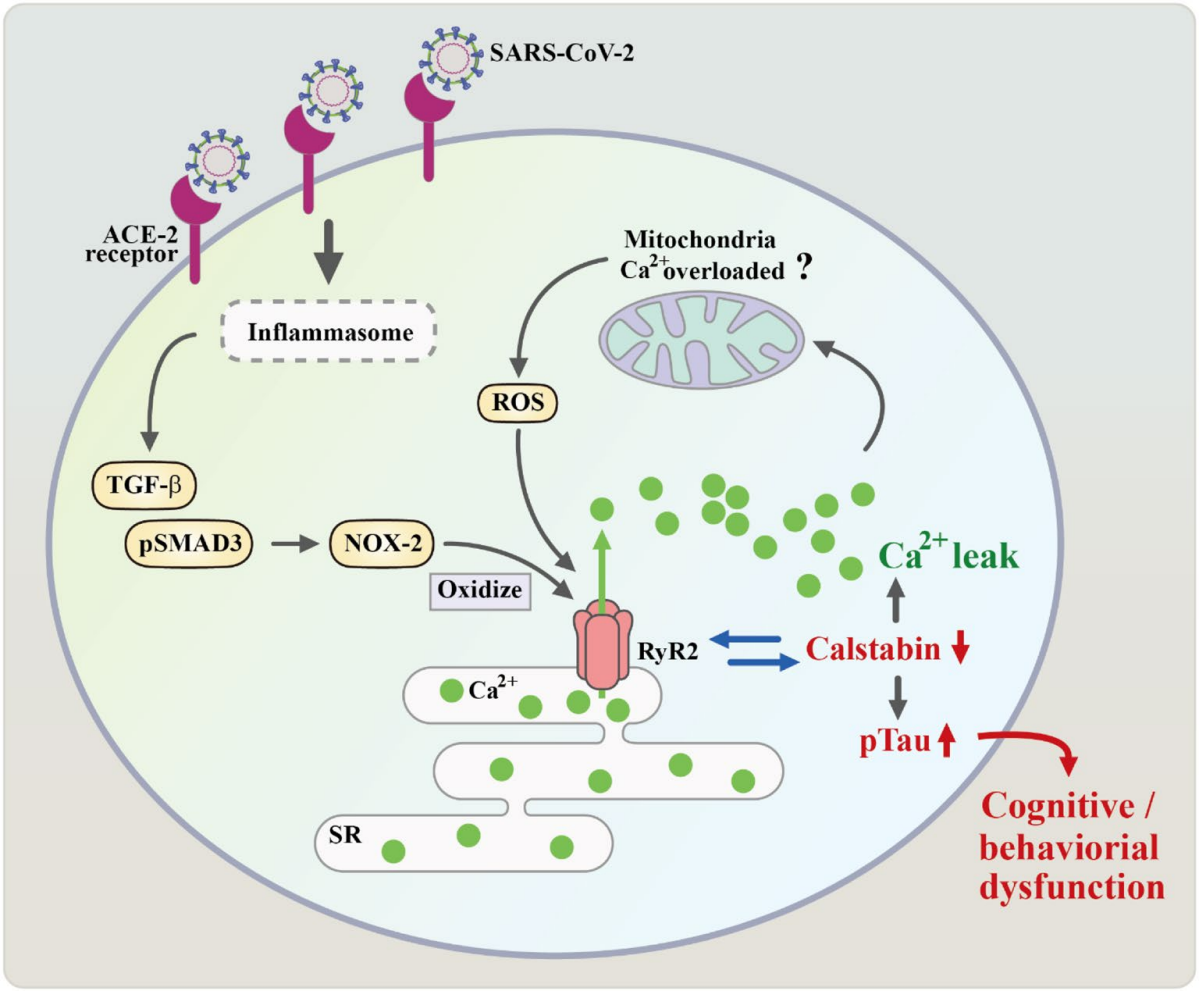
Mechanism of leaky ryanodine receptor 2 channels in cognitive impairment after SARS‐CoV‐2 infection. A cartoon illustration offers a concise explanation of the potential mechanism underlying leaky ryanodine channels, which regulate cellular calcium overload and contribute to cognitive impairment in individuals affected by SARS‐CoV‐2.

SARS‐CoV‐2 infection can result in the activation of leaky RyR2 channels, which may contribute to the observed cardiac, pulmonary, and cognitive dysfunction [52]. Meanwhile, inadequate regulation of intracellular calcium via leaky ryanodine receptor 2 (RyR2) channels and the activation of Alzheimer's disease (ad)‐like neuropathology (hyperphosphorylation of tau) have been associated with neurological symptoms, particularly the ‘brain fog’ observed in long COVID [52]. When SARS‐CoV‐2 enters cells via the ACE2 receptor, it induces a stress response, either via the inflammasome or by activating TGF‐β pathways. Decreased ACE2 expression results in increased fibrosis in the lungs and overactivation of inflammatory pathways, including TGF‐β. Increased TGF‐β stimulates SMAD3 (pSMAD) and promotes the expression of NADPH oxidase 2 (NOX2). Increased expression of NOX2 under stress could lead to the channel oxidation of leaky RyR2 channels and depletion of the channel‐stabilizing protein calstabin, leading to channel leakage of calcium from the endoplasmic or sarcoplasmic reticulum (SR). This can cause mitochondrial malfunction due to calcium overload [53]. As an intriguing piece of evidence, patients with SARS‐CoV‐2 infection have shown decreased expression of calstabin‐2 in the brain and cerebellum, rendering them more vulnerable to cytosolic calcium overload [52]. The observation of malfunctioning leaky RyR2 channels in SARS‐CoV‐2 infected individuals, in conjunction with the established role of neuronal RyR2 remodeling in cognitive dysfunction induced by physiological stress [54], strongly suggested the potential contribution of dysregulated RyR2 signaling in the symptoms of cognitive impairment following SARS‐CoV‐2 infection (Figure 4).

#### 1.4.6 Vasculature Dysfunction

**FIGURE 5:**
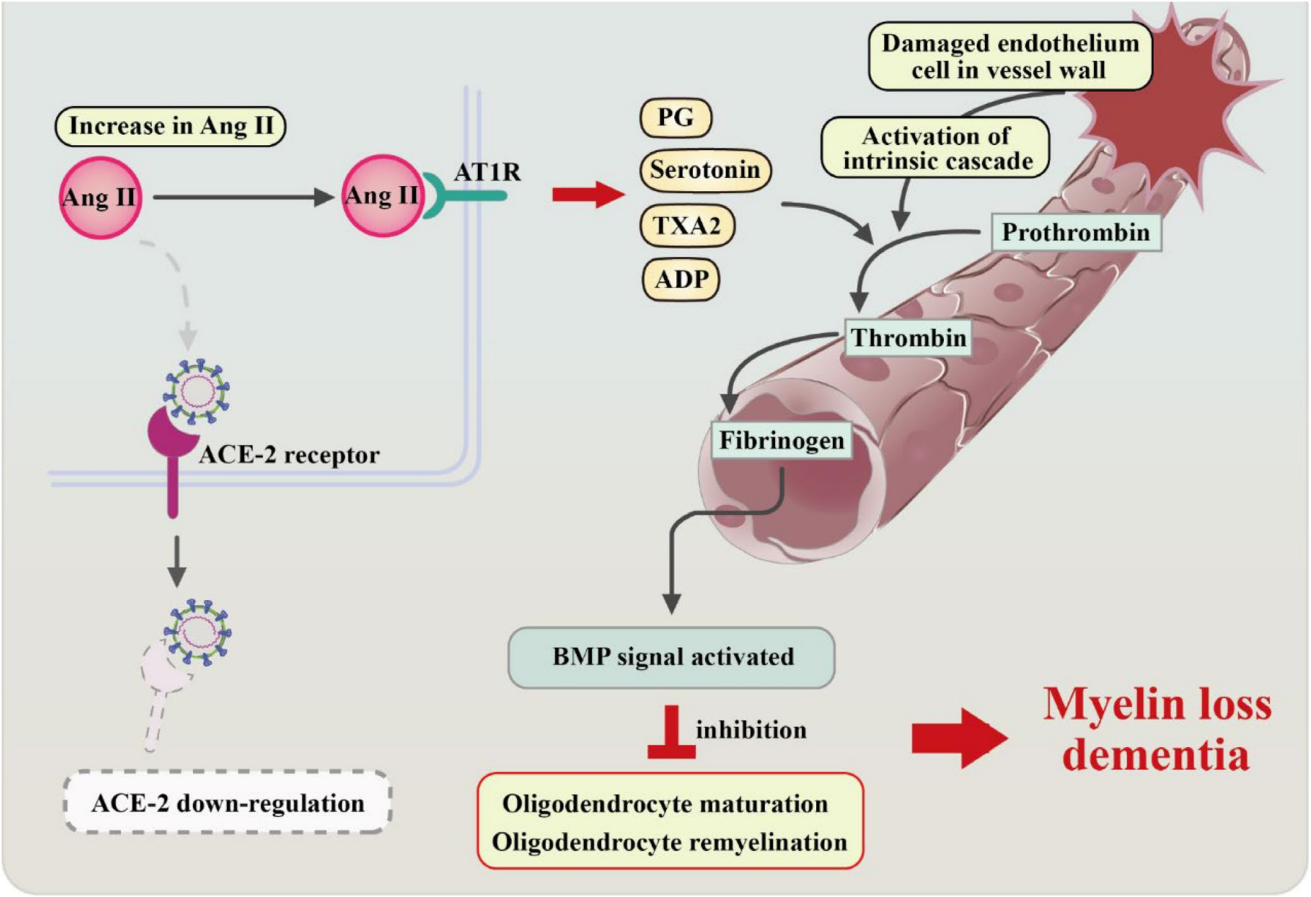
Vascular dysfunction and cognitive impairment in COVID‐19 patients. A cartoon figure demonstrates (1) the cascade of events triggered by endothelial damage, leading to the activation of the intrinsic pathway and bone morphogenetic protein (BMP) activation. (2) The consequences of decreased ACE2 receptor levels, resulting in increased Angiotensin II (Ang II) levels and the release of vasoactive compounds such as prostaglandins (PG), thromboxane (TXA2), serotonin, and adenosine diphosphate (ADP). These factors collectively contribute to additional endothelial injury, which may potentially lead to cognitive impairment in individuals with COVID‐19.

For the maintenance of cognitive function, the integrity of cortical white matter is crucial. COVID‐19 infected cases have reported the susceptibility of cerebral white matter to variations in cerebral blood flow [55]. Vascular dysfunction in COVID‐19 patients can lead to CI through three possible mechanisms:

- *Small Vessel Disease* (SVD): BBB leakiness and damaged endothelial cells and pericytes contribute to SVD‐associated brain injury. Endothelial dysfunction and pericyte loss are intricately associated with the influx of toxic plasma components, notably fibrinogen. This influx, in turn, triggers the activation of the bone morphogenetic protein (BMP) signaling pathway, subsequently hindering oligodendrocyte maturation and impeding the process of remyelination [46].
- *Thromboembolism*. Thromboembolic occlusion of the cerebral blood vessels can result in various neurological dysfunctions, encompassing CI and dementia. The acute dysregulated inflammatory response observed in COVID‐19 patients, aimed at eliminating the infectious agent, can lead to cerebral ischemia by causing hypercoagulability and vasculopathy in cerebral blood vessels. Endothelial cells, stimulated by immune cell infiltrates into brain tissue and an increase in proinflammatory cytokine production, trigger the coagulation cascade, potentially leading to the formation of microthrombi and subsequent cerebral ischemia [36].
- *Activation of coagulation cascade*. Based on the evidence of obtained by the studies for SARS‐CoV‐1, a hypothesis for SARS‐CoV‐2 has been proposed [56]. As we discussed in the section of RAS, a balanced, lower ACE/ACE2 ratio within the vascular endothelium inhibits the activation of prothrombotic cascade by converting Ang II to Ang 1–7. At the same time antithrombotic effects are also enhanced with binding of Ang 1–7 to MasR [57]. However, in COVID‐19‐infected patients, the binding of the virus to ACE2 suppresses ACE2 expression levels, leading to an imbalance in the ACE/ACE2 ratio and promoting tissue injury. This imbalance results in Ang II binding to AT1 receptors, initiating the prothrombotic cascade and potentially leading to vessel thrombosis [58, 59, 60]. Additionally, the kallikrein‐bradykinin pathway may be activated in vascular endothelium due to ACE2 suppression, leading to platelet aggregation and thrombosis [56] (Figure 5).

#### 1.4.7 Kynurenine Pathway

The Kynurenine Pathway (KP) is a metabolic route responsible for the degradation of tryptophan, a crucial amino acid. This pathway involves enzymes such as indoleamine 2,3‐dioxygenase 1 and 2 (IDO1/2) and tryptophan 2,3‐dioxygenase (TDO), which convert tryptophan into N‐formyl‐L‐kynurenine [61]. Subsequently, N‐formyl‐L‐kynurenine is transformed into L‐kynurenine (L‐KYN). This L‐KYN can take two main pathways, either conversion into kynurenic acid (KYNA) via kynurenine aminotransferases (KAT) or into 3‐hydroxykynurenine (3‐HK) through kynurenine 3‐monooxygenase (KMO). The latter compound, 3‐HK, can further proceed into quinolinic acid (QUIN), which can ultimately contribute to the production of the essential cellular cofactor, NAD+ (nicotinamide adenine dinucleotide), which plays a critical role in cellular metabolism [62].

**FIGURE 6:**
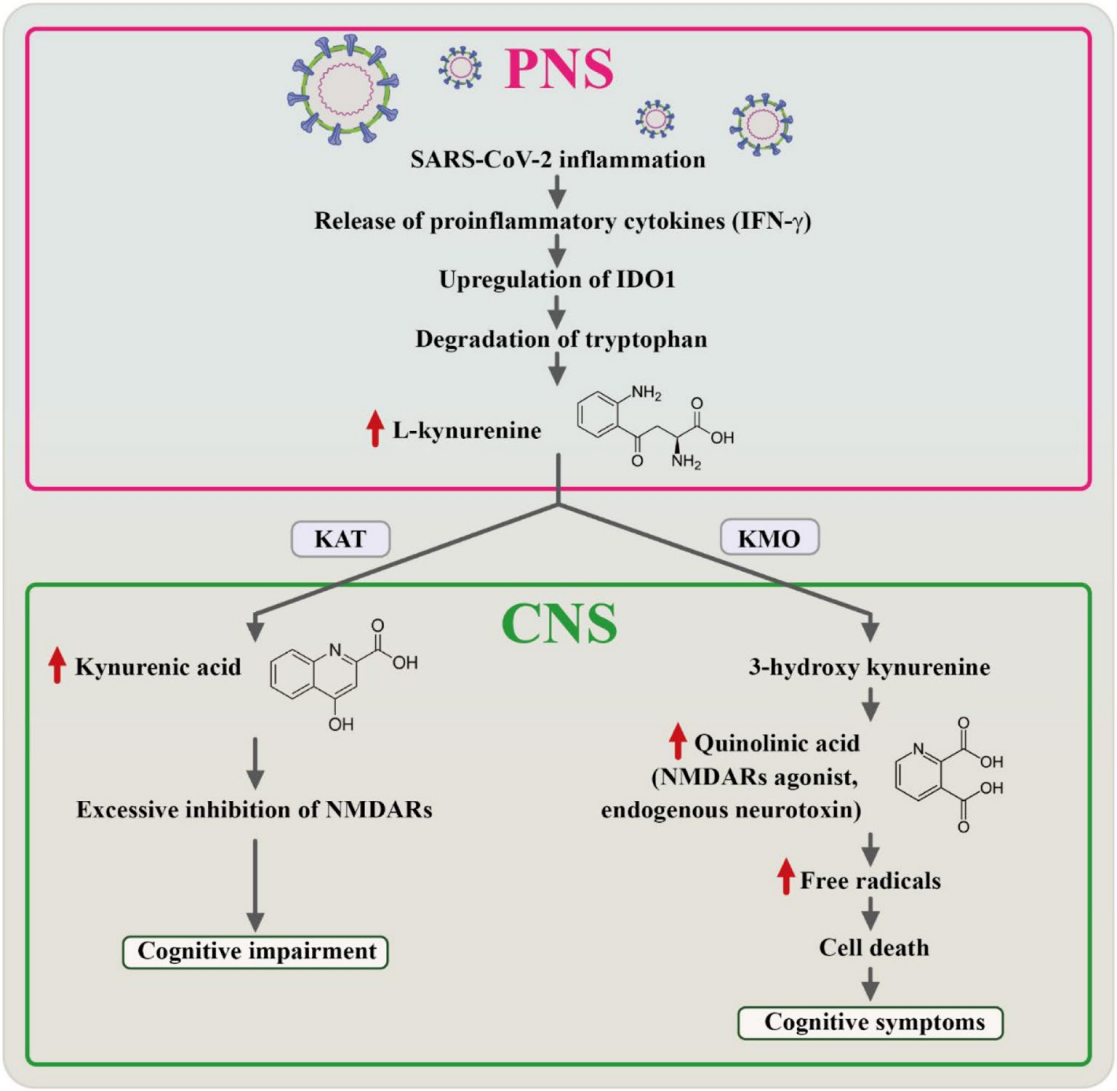
Impact of SARS‐CoV‐2 on the kynurenine pathway in the peripheral and central nervous system which leads to cognitive impairment. A visual representation depicts the impact of SARS‐CoV‐2 on the kynurenine pathway in both the peripheral and central nervous systems, potentially leading to cognitive impairment or cognitive symptoms following SARS‐CoV‐2 infection.

Accumulating evidence indicates that these KP metabolites play a dual role in modulating both inflammatory responses and neurological functions. Remarkably, patients infected with COVID‐19 have exhibited altered KP profiles [63], characterized by lower levels of tryptophan and higher concentrations of L‐KYN in their serum compared to control subjects [64]. Within the peripheral nervous system, monocytes activate the KP by upregulating the expression and activity of IDO1 in response to proinflammatory cytokines such as interferon‐gamma (IFNγ). This activation leads to the increased breakdown of tryptophan into L‐KYN. Importantly, L‐KYN can traverse the BBB and enter the CNS, where it undergoes conversion into KYNA. KYNA, in general, acts as a neuroprotective agent, safeguarding neuronal cells by inhibiting NMDA receptors (NMDARs), α7 nicotinic acetylcholine receptors (α7nAChRs), and preventing excessive glutamate‐induced excitotoxicity [65, 66]. However, excessively high levels of KYNA can have detrimental effects, inducing excessive inhibition of NMDARs, which may contribute to the observed CI [67] (Figure 6). Furthermore, in patients with COVID‐19, increased KMO activity due to proinflammatory cytokines within CNS cells, such as microglia and infiltrating macrophages, promotes the formation of the neurotoxic QUIN [68, 69]. QUIN, functioning as an NMDAR agonist, can initiate oxidative cell death and neuronal apoptosis at elevated concentrations, potentially exacerbating CI and encephalopathy in certain COVID‐19 patients [67] (Figure 6).

#### 1.4.8 Psychological Distress

Cognitive deficits are characteristic features of various mental disorders [31]. The practice of isolating COVID‐19 infected individuals and implementing social distancing to prevent the spread of pandemics has raised concerns about the heightened risk of mental health issues. These mental health challenges or psychological distress may progressively contribute to cognitive decline and potentially elevate the risk of various neurodegenerative diseases. One of the potential drivers of CI in COVID‐19 patients elicited by psychological distress is low‐level or mild chronic inflammation, which is associated with the release of proinflammatory cytokines. Such inflammatory response can activate microglial and astroglial cells while diminishing the availability of neurotransmitters such as monoamines at the synapses. Activated microglia can further lead to a decrease in BDNF production and inhibit neurogenesis, ultimately contributing to neurodegeneration [70]. Psychosocial stress plays a pivotal role in this context, with the hippocampus serving as both a target and the main regulator of the brain's response to stress. Hippocampal neurons possess glucocorticoid receptors and inhibitory afferents that help regulate the release of hypothalamic corticotropin‐releasing factor (CRF). Prolonged exposure to elevated cortisol levels (hypercortisolemia) due to chronic stress can lead to neuronal damage and cell death in the hippocampus, resulting in hippocampal atrophy and CI [71]. This interplay between chronic stress, neuroinflammation, and cognitive function underscores the importance of addressing psychological distress in the context of COVID‐19 and its potential long‐term cognitive consequences.

### 1.5 CI in the Acute Phase of COVID‐19

CI in the context of COVID‐19 can significantly affect an individual's ability to think, learn, remember, make judgments, and solve problems [72]. A meta‐analysis reported that executive function, memory, and attention (cognitive domains) exhibited the highest degree of impairment, with concentration difficulties identified as the major symptom in a recent survey [73, 74]. Additional meta‐analyses revealed that brain fog constituted approximately 32%, whereas attention and memory impairments accounted for 22% and 17.5%–35%, respectively [75]. Early detection of cognitive symptoms during the course of COVID‐19 may be essential for identifying patients at a higher risk of developing cognitive deficits. Tolentino et al. [76] conducted mental scoring and objective attention tests on a patient who initially presented with attentional deficits while driving, later experiencing symptoms such as fever, ageusia, and anosmia. It was observed that the patient's attention levels decreased until day 10, after which the issue of attention gradually improved and returned to normal by day 16. This case suggests that attentional deficits could serve as an initial symptom preceding respiratory impairments in patients infected with COVID‐19 [76]. A nationwide study in the UK revealed that altered mental status, including encephalopathy, encephalitis, and primary psychiatric diagnoses, is the second most common presentation among younger individuals with COVID‐19 [77]. During the acute phase of COVID‐19, the incidence of CI has been reported to vary, ranging from 61.5% to 80% in hospitalized patients with mild to moderate severity and reaching up to 80% in patients with moderate to severe illness, especially those in rehabilitation clinics [78]. A biobank study comparing brain scans of patients before and after COVID‐19 infection demonstrated a significant cognitive decline associated with the structural atrophy of crus II, a cognitive lobule of the cerebellum [79]. Furthermore, a systematic review conducted by Tavares‐Junior et al. unveiled that CI was evident even in cases of mild to moderate COVID‐19, with higher rates observed among patients in the postacute phase [80]. These findings underscore the importance of recognizing and addressing cognitive symptoms in the acute phase of COVID‐19, as they may have lasting consequences for patients' cognitive function.

### 1.6 CI in Long‐COVID‐19

Prolonged cognitive symptoms or the exacerbation of existing ones in COVID‐19 patients are often triggered by intense cognitive activity [81]. These persistent cognitive deficits are more frequently observed in patients who experienced severe COVID‐19 infections, even if they did not manifest distinct neurological symptoms during the acute phase [77]. Importantly, the severity of cognitive symptoms is significantly associated with the duration of the disease, with patients experiencing longer durations of the disease being more prone to developing cognitive deficits associated with long‐COVID [82]. Cognitive dysfunction ranks as the second most retained symptom after fatigue in long‐COVID patients [83]. The prevalence of CI varies, with reports indicating rates of 54% in moderate cases and 65% in severe cases [78]. A study conducted on COVID‐19 recovered patients 6 months post‐discharge found that 18% of these individuals, among the 40% who showed neurological abnormalities, manifested cognitive deficits [16]. Furthermore, even among non‐hospitalized COVID‐19 patients (mild to moderate cases) who presented to the Neurology COVID Clinic due to persisting symptoms after 6 weeks, approximately 80% reported experiencing brain fog [84]. Other cognitive issues, such as memory problems and speech and language difficulties, were more frequently reported after 8 weeks [85]. Meanwhile, a recent meta‐analysis further highlighted that verbal memory impairments and semantic verbal fluency are the most affected cognitive domains in long‐COVID patients [78]. In a recent one‐year longitudinal study involving older COVID‐19 survivors, severe cases showed an increased risk of early onset, late onset, and progressive cognitive decline, whereas non‐severe cases were at higher risk of early onset cognitive decline, even after adjusting for risk factors [86]. It is important to note that COVID‐19 patients with CI may also experience delirium [78]. A recent review by Hawkins et al. found that over half of the hospitalized COVID‐19 patients exhibited delirium [87]. The World Health Organization (WHO) has emphasized that alteration in consciousness could be one of the most critical signs of COVID‐19. Additionally, previous literature has shown a significant overlap between CI and depressive symptoms.

Here we have compiled a list of disparities in CI observed during either the acute or postacute phases of COVID‐19 as Table 1.

**TABLE 1:** Cognitive deficits in acute and postacute phases of COVID‐19.

Aspect	Acute Phase of COVID‐19	Long COVID
Presence of cognitive deficits	May or may not be present in all acute COVID‐19 patients	Cognitive deficits form a core aspect of long COVID, may also occur devoid of other organ involvement
Literature on cognitive deficits	Higher percentage of cognitive deficits in severe COVID‐19 cases; however, recent literature explains cognitive defects in mild–moderate cases also	Cognitive impairment is not dependent on COVID‐19 severity, but is affected by pre‐existing neurological disorders
Prevention of CI symptoms	CI symptoms cannot be prevented once the virus enters the brain	CI can be prevented by non‐pharmacological treatment if recognized earlier in the acute phase
Improvement timeline	Few reports suggest improvement in cognition by the 10th day of onset of the disease; otherwise, symptoms may deteriorate with time	Improvement possible only with rehabilitation and treatment
Rehabilitation impact	Inpatient rehabilitation can change cognitive improvement scores	Requires long‐term rehabilitation with cognitive training

## 2 Management of Acute and Long‐Term CI

Treatment for CI associated with COVID‐19 can be categorized into three major approaches, as outlined in Table 2.

**TABLE 2:** List of modes of treatment for CI in COVID‐19.

Category	Subcategory	Treatment	References
(I) Pharmacological Treatment	1. Anti‐inflammatory therapy	(a) TNF‐ɑ Inhibitors (b) NSAID (c) IL‐1R Inhibitor – Anakinra (d) IL‐6 Inhibitor—Siltuximab (e) IL‐6R Inhibitor—Tocilizumab, Sarilumab	[32] [88] [36] [36] [36]
	2. RAS targeting drugs	(a) AT1 receptor antagonist—Losartan (b) ACEI—Perindopril (c) ACE2 activator (DIZE) and rhACE2 (d) RAS peptides—Ang 1–7	[89] [89] [90, 91] [92, 93]
	3. Targeting NLRP3 inflammasome	(a) MCC950 (b) BAY11‐7082 (c) OLT1177 (d) JC124 (e) VX765 (f) Colchicine	[94] [95] [96] [97] [98] [99, 100, 101]
	4. Therapy against viral entry pathways	(a) ACE‐2 inhibitors (b) VEGF‐A/NRP1 blocker	[102] [103, 104]
	5. Targeting RYR2	(a) Rycal	[52]
	6. KP Pathway Inhibition	(a) Peripheral KMO inhibitors (b) KAT II Inhibitors (c) Brain Permeable KMO inhibitor	[105, 106] [107] [106]
(II) Nutraceuticals		1. Curcumin 2. Resveratrol 3. Quercetin 4. Ginseng	[108, 109, 110, 111, 112] [108, 109, 113, 114] [115, 116] [108, 117, 118]
(III) Non‐Pharmacological Treatment		1. Noninvasive brain stimulation 2. Rehabilitation 3. Cognitive training	[119, 120, 121, 122, 123] [124, 125, 126, 127]

### 2.1 Pharmacological Treatment

#### 2.1.1 Anti‐Inflammatory Therapy

Since inflammation plays a role in CI, anti‐inflammatory therapeutics are crucial in preventing long‐term cognitive deficits in COVID‐19 patients [128]. For example, TNF‐α inhibitors have shown promise in reducing neurofibrillary tangles and amyloid protein, as seen in animal studies [32]. A single peripheral etanercept (TNF inhibitor) dose was linked in a case study with improved executive functions and verbal fluency [129]. The combination of α2A‐adrenoceptor agonist guanfacine and antioxidant N‐acetylcysteine (NAC) has shown promising results in treating brain fog and related cognitive problems. Guanfacine strengthens prefrontal cortical connections, which are necessary for functions including working memory and concentration. Conversely, NAC restores intracellular glutathione levels, therefore shielding neurons from oxidative stress that is often raised in Long COVID‐19 [130]. Additionally, Kondo et al. [131] demonstrated that gradual administration of extended‐release guanfacine (GXR) 2–4 mg/day in a patient with long COVID improved working memory and attention issues. Citicoline may treat COVID‐19‐related cognitive decline by inhibiting phospholipase 2 and upregulating sirtuin1 protein levels in the brain, improving neuronal plasticity and cognitive function [132]. Treatment with citicoline 1000 mg/daily led to a marked improvement in symptoms after 6 months in a 59‐yearyear‐old nonhospitalized COVID‐19 patient [133]. It may also repair mitochondrial dysfunction and reduce oxidative damage. When considering the suppression of cytokine release, targeting ILs such as IL‐1 and IL‐6 becomes crucial; drugs such as Anakinra (monoclonal antibodies (mAbs)), which target IL‐1R, as well as Tocilizumab and Sarilumab, which target IL‐6R, have the potential to alleviate neurotoxicity associated with cytokine release syndrome [36, 134]. However, the cost of these valuable mAbs can be a significant burden on patients and healthcare systems [38].

#### 2.1.2 RAS Targeting Drugs

The neuroprotective effects of ACE inhibitors (ACEIs) and angiotensin receptor blockers (ARBs) are predominantly attributed to their modulation of the ACE2/Ang‐(1–7)/Mas axis, resulting in their anti‐inflammatory responses. Extensive studies conducted in animal models of progressive neurodegenerative disorders have greatly confirmed such neuroprotective properties. For example, the administration of AT1R antagonists like losartan or ACEIs such as perindopril in Parkinson's disease models has shown promising beneficial effects. These interventions elevated striatal dopamine levels, improved neuronal survival, and reduced motor dysfunction [89]. Similarly, the utilization of the ARB telmisartan in an experimental Alzheimer's disease mouse model yielded cognitive enhancements, increased cerebral blood flow, and mitigated inflammation and oxidative stress within the mouse brain [89]. Recent studies have also explored the neuroprotective effects of ACE2 stimulation. The ACE2 activator, DIZE, administered intraperitoneally to Tg2576 mice, successfully restored cognitive function [90]. Moreover, recombinant ACE2 (rhACE2) has demonstrated a significant affinity for binding to the spike protein of the SARS‐CoV‐2 virus, inhibiting its attachment to host cells in vitro [91]. Additionally, a combination therapy involving remdesivir and ACE2 has demonstrated enhanced antiviral effects in an in vitro study [135]. However, the efficacy of this treatment strategy in ARDS remains contentious. Evidence suggests that such treatment strategy has proven to be rather ineffective in reducing Ang II production or increasing RAS peptides Ang‐(1–7) and Ang‐(1–5), yet it did not lead to the alleviation of symptoms or a reduction in disease severity. Pulmonary function has shown little difference between the treatment and control groups [92]. Conversely, exogenous administration of Ang‐(1–7) has demonstrated improved clinical outcomes in severe ARDS cases by enhancing anti‐inflammatory responses via MasR activation [92]. For instance, intracerebrovascular administration of Ang‐(1–7) over 4 weeks significantly improved cognition and cerebrovascular reactivity in animal models of Alzheimer's disease [93]. Currently, numerous phase I and II clinical trials are underway to evaluate the safety and efficacy of Ang‐(1–7) administration. In a case study, Cholytilin (choline alfoscerate), administered at a dosage of 800 mg in the morning and 400 mg at lunchtime used in 50 participants with post‐COVID CI, led to complete regression of CI in 74% of participants [136].

#### 2.1.3 Targeting NLRP3 Inflammasome

Several preclinical models suggest that agents inhibiting the NLRP3 inflammasome, a key player in neuroinflammation, hold promise for diseases driven by neuroinflammatory mechanisms. These small chemical compounds possess the advantage of crossing the BBB, rendering them valuable in conditions such as CI [137]. Although the therapeutic efficacy of specific and non‐specific NLRP3 inhibitors in treating CI is yet to be fully evaluated, they have shown promise in severe COVID‐19 infected cases. NLRP3 inhibitors reduce inflammation by limiting the activity of caspase‐1, resulting in reduced secretion of inflammatory markers such as IL‐1β [138, 139].

- MCC950: MCC950 is a specific NLRP3 inflammasome inhibitor known for treating complications affecting the lungs, cardiovascular system, and nervous system arising from NLRP3 activation [138, 140]. In mouse models of spinal cord injury, MCC950 demonstrated the ability to reduce inflammation and improve neurological outcomes. Exploring the potential of MCC950 in managing neuroinflammatory symptoms secondary to SARS‐CoV‐2 infection is warranted [94].
- BAY11‐7082: BAY11‐7082 is an irreversible NFκB inhibitor that inactivates NLRP3 by alkylating its cysteine groups [92, 141]. This compound prevented SARS‐CoV‐2 S1‐induced NLRP3 inflammasome stimulation, reducing the production of pro‐inflammatory mediators and subsequently mitigating COVID‐19‐associated neuroinflammation [95].
- OLT1177: also known as Dapansutrile, OLT1177 is an oral β‐sulfonyl nitrile agent specifically targeting the NLRP3 inflammasome [138]. In a transgenic mouse model of Alzheimer's disease (ad), OLT1177 demonstrated the ability to enhance synaptic plasticity, reduce microglial activation, and decrease Aβ plaque burden. Its potential in reducing neuroinflammation in the context of COVID‐19 is under investigation [96].
- JC124: JC124, a specific NLRP3 inhibitor, has shown promising results in the treatment of CI for neurodegenerative disorders. In a study on ad‐related pathologies, JC124 administration in CRND8 APP transgenic mice reduced both soluble and insoluble Aβ levels, decreased APP cleavage, enhanced astrocytosis, and reduced microglial activity. JC124 also decreased oxidative stress, confirming its neuroprotective effects [97].
- VX‐765: VX‐765 is a potent and selective inhibitor of caspase‐1 that suppresses the activation of the inflammasome. Administration of VX‐765 to mouse models with spinal cord injury resulted in reduced caspase‐1, IL‐1, and IL‐18 secretion. This intervention also improved functional recovery by alleviating neuronal injury and demyelination of white matter [98].
- Colchicine: colchicine, a tricyclic alkaloid, is currently used for managing conditions such as familial Mediterranean fever, gouty arthritis, and pericarditis [99]. Given its effectiveness in CNS‐related pathologies such as neuro‐Behçet's syndrome [100] with its well reported efficacy in the suppression of NLRP3 inflammasome [142, 143], colchicine holds great potential for the management of COVID‐19‐associated neuroinflammation [144]. Recent studies have demonstrated that increasing doses of colchicine significantly reduce mortality in COVID‐19 inpatients [145]. Specifically, colchicine loading doses of 4 mg were found to be more effective than 2 mg, achieving an overall relative risk reduction of 70.7%. Inpatients treated with colchicine experienced markedly lower mortality rates compared to standard‐of‐care (SOC) patients (5.7% vs. 19.53%). Such evidence underscores colchicine's potential as a therapeutic option for mitigating COVID‐19‐associated neuroinflammation.
- Ac‐YVAD‐cmk: administering Ac‐YVAD‐cmk to mouse models of depressive‐like disorders inhibited immune activation and NLRP3 activity by blocking caspase‐1 [101]. This compound has shown promise in combating neuroinflammation and multiorgan damage in COVID‐19 patients by selectively inhibiting IL‐1β converting enzyme [138].

#### 2.1.4 Therapy Against Viral Entry Pathways

Current investigations aimed at averting viral entry are concentrated on the inhibition of critical mediators, notably ACE2 and NRP‐1, which elicit diverse physiological responses. Although ACEIs have exhibited modest improvements in conjunction with the therapy for COVID‐19 patients relative to ARBs, their efficacy as a monotherapy remains comparatively less pronounced [102]. The cytokine storm characteristic of this context often perturbs the integrity of the BBB, allowing cytokines to enter the CNS. Mechanistically, furin cleavage substrates of the SARS‐CoV‐2 spike protein interact with the vascular endothelial growth (VEGF) binding site (VEGF‐A) on NRP‐1. This intricate VEGF‐A/NRP‐1 interaction has garnered significant attention within the field of cancer therapy research [103]. To mitigate such phenomena, the intervention of VEGF signaling via a peptide that blocks NRP‐1 has demonstrated a notable restorative impact on the compromised BBB. Therefore, a therapeutic agent capable of inhibiting the VEGF‐A/NRP1 pathway might serve as a means to impede the entry of the SARS‐CoV‐2 virus into healthy cells, addressing the cascade of events encompassing cellular entry, cytokine storm, and CNS damage. In this regard, targeting NRP1 with a blocking agent stands out as a promising and valuable approach [104].

#### 2.1.5 Targeting RyR2 Channels

Recent research has proposed that leaky RyR2 channels could be valuable targets for treating cognitive decline in long‐term COVID‐19 infection [52]. Specifically, Rycal drugs have emerged as potential solutions, as they are designed to restore the integrity of these leaky RyR2 channels by repairing calstabin2 binding and stabilizing their closed state. As a result, the therapeutic targeting of these leaky RyR2 channels holds significant clinical promise in alleviating the persistent cognitive deficits associated with long COVID‐19 [52, 146].

#### 2.1.6 KP Pathway Inhibition

Recent studies have identified the activation of the KP in individuals affected by COVID‐19 [147]. Therefore, the modulation of the KP pathway emerges as a promising therapeutic avenue for mitigating CIs associated with COVID‐19 infection.

**FIGURE 7:**
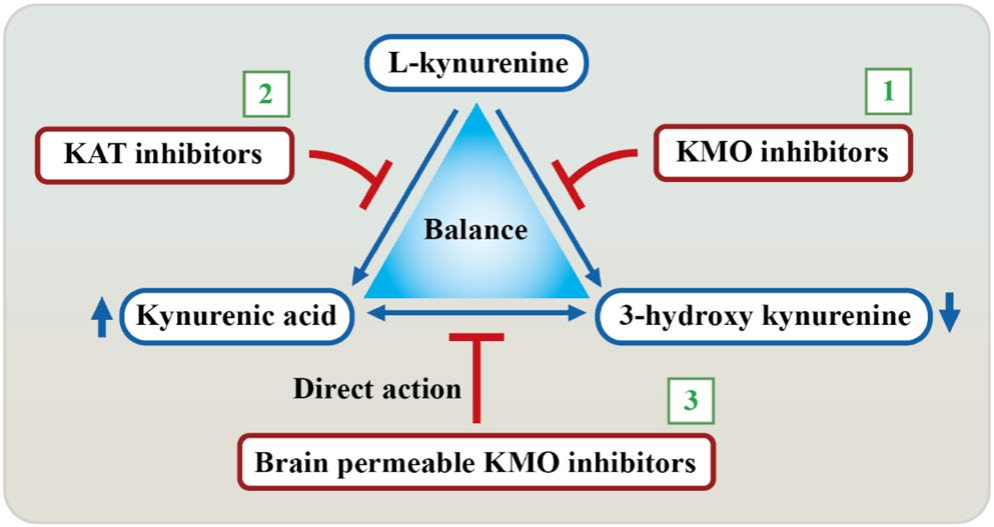
Mechanisms for ameliorating cognitive impairment caused by elevated kynurenine metabolites. A cartoon figure demonstrates three mechanisms for inhibiting cognitive impairment caused by elevated kynurenine metabolites: (1) KMO inhibitors, (2) KAT inhibitors, and (3) Brain‐permeable KMO inhibitors.

In general, inhibitors of KMO, an enzyme in the KP pathway, can lead to an increase in kynurenic acid (KYNA) levels. This, in turn, has been associated with a reduction in neuronal damage and improvements in cognitive function in animal models [105]. Notably, COVID‐19 patients have been found to exhibit elevated peripheral KYNA levels, which may reflect the altered KP pathway in the CNS [148]. However, it is important to note that KMO inhibition, while holding promise for modulating the immune response, may initially have adverse effects on cognitive function. Elevated KYNA concentrations could hinder neurotransmitter signaling critical for memory and learning. Nevertheless, the reduction of inflammation and proinflammatory cytokines resulting from KMO inhibition could, over time, contribute to the normalization of the KP pathway. Conversely, inhibitors of kynurenine aminotransferase II (KATII), which act to block the conversion of L‐kynurenine (L‐KYN) into KYNA, offer a distinct avenue for reducing cognitive deficits in COVID‐19 patients by directly lowering KYNA levels [107]. An additional advantage lies in the recent development of brain‐permeable KMO inhibitors, which directly address the production of detrimental KP metabolites within the CNS [106]. As a result, this approach may significantly curtail the accumulation of neurotoxic substances such as 3‐hydroxykynurenine (3‐HK) and quinolinic acid (QUIN), thereby enhancing the neuroprotective effects of KYNA within the CNS (Figure 7).

### 2.2 Nutraceuticals

The term “nutraceutical”, first introduced by Dr. Stephen DeFelice, seamlessly merges the concepts of ‘nutrient,’ derived from dietary sources including food and herbs, with ‘pharmaceutical agent,’ typically associated with drugs. It serves as a descriptor for substances that offer protective properties against various diseases [149]. Recent research suggests that nutraceutical agents have the potential to modulate the expression of various mRNAs and microRNAs (miRs) that play important role in cytokine regulation in the context of various inflammatory conditions [108]. However, their clinical efficacy and utility remain inconclusive due to variations in bioavailability, dosage, and standardized preparations [109].

#### 2.2.1 Curcumin

Curcumin, a natural compound derived from the rhizomes of *Curcuma longa* and found in turmeric and other plants like ginger, has garnered considerable attention for its potential in addressing CI in individuals affected by COVID‐19.

Curcumin has demonstrated its capacity to inhibit the production of Aβ fibrils and to destabilize pre‐existing Aβ fibrils, which are known for their neurotoxic properties [109]. Additionally, in the realm of animal studies, the prophylactic administration of curcumin to rats injected with Aβ4 and Aβ42, both of which contribute to neurodegeneration and Aβ deposits, yielded substantial improvements in memory function [110]. Belviranli and colleagues reported a remarkable enhancement in spatial memory in aged female rats following a 12‐day regimen of curcumin supplementation [150]. Furthermore, in a randomized controlled trial involving healthy elderly participants, the administration of 400 mg of Longvida, an optimized curcumin, resulted in significant enhancements in working memory and mood [111]. Nonetheless, investigations using Biocurcumax, a standardized curcumin preparation, to avert CI showed no discernible differences in cognitive measures, except in the context of treatment duration [151]. In in vitro experiments, the administration of curcumin to LPS‐treated BV2 microglial cells resulted in a notable reduction in the expression of inflammatory mediators, including TNFα and IL1β, via enhanced miR‐199b‐5p levels [112]. These findings underscore the potential of curcumin as a nutraceutical for mitigating CI in individuals affected by COVID‐19. Nonetheless, further research is imperative to establish standardized dosages and preparations, unlocking its complete therapeutic benefits.

#### 2.2.2 Resveratrol

This class of polyphenolic compounds exhibits pleiotropic protective effects with potential clinical value in the prevention and management of neurodegenerative disorders [109]. RSV exerts multifactorial biological effects both in vitro and in vivo, including the inhibition of Aβ aggregation, reduction of oxidative stress, suppression of brain pro‐inflammatory mechanisms, and prevention of neuronal cell death [113]. In BV‐2 microglial cells, RSV significantly reduces the production of inflammatory mediators induced by LPS, such as TNF‐α, IL‐1β, and IL‐6, through the upregulation of miR‐146a‐5p [114]. Remarkably, RSV, administered within the concentration range of 25–250 μM, has demonstrated anti‐inflammatory effects in the context of severe COVID‐19 infections by modulating crucial cytokines, namely IL‐1β, IL‐6, and TNF‐α [108]. A study conducted by Witte and colleagues achieved notable memory retention in elderly subjects through the utilization of a combination of resveratrol (RSV, 200 mg/day) and quercetin (300 mg/day) [152]. Moreover, when supplemented with 5% quercetin and 5% rice bran phytate in the Longevinex formulation, resveratrol may potentially exhibit increased bioavailability. Nevertheless, it is worth noting that this hypothesis remains unsupported by clinical research to date [109]. These findings suggest that specific formulations of resveratrol supplementation could enhance clinical efficacy in mitigating CI in individuals affected by COVID‐19; additional research is essential to validate this possibility.

#### 2.2.3 Quercetin

Quercetin, a member of the flavonoid family, is abundantly found in vegetables and fruits (4–79 mg/100 g). Due to its relatively low bioavailability, the effective dose varies considerably. Quercetin is renowned for its natural enhancement of the body's immune response and innate defense mechanisms against chronic diseases. In a study conducted by Galleggiante and colleagues, the treatment of lipopolysaccharide‐stimulated bone marrow‐derived dendritic cells (BMDCs) with quercetin significantly increased miR‐369‐3p expression levels, resulting in a notable reduction in mRNA and protein levels of TNF‐α and IL‐6 [115]. Quercetin has demonstrated effective anti‐inflammatory properties induced by IL‐1 and TNF‐α in vitro microglial cells at concentrations of 10 and 100 μM [116].

#### 2.2.4 Ginseng

Ginseng, derived from the roots of *Panax ginseng* , has a long‐standing reputation in both traditional and modern medicine as a health supplement. Ginsenosides, steroid‐like saponin compounds found in ginseng extract, have been recognized for their ability to enhance normal physiological processes and address inflammation in various diseases, including cancer, diabetes, and conditions related to neuronal and cardiovascular health [108].

Furthermore, another notable bioactive compound within ginseng extract, Compound K, has demonstrated its capability to inhibit NLRP3 inflammasomes and contribute to the mitigation of neuronal and cognitive decline. In addition to this, a range of bioactive agents found within various *Panax* species have also been identified as effective inhibitors of NLRP3 inflammasome activation and agents for reducing neuroinflammation in both macrophages and neurons [117, 153]. In an in vitro study conducted by Gao and colleagues, ginsenoside Rg1 demonstrated noteworthy anti‐inflammatory properties in LPS‐induced activation of BV2 microglial cells and ventral mesencephalic primary microglial culture. At a concentration of 10 μM, it effectively reduced the mRNA and protein levels of TNFα, IL‐1β, iNOS, and COX‐2 [118]. These findings suggest that regular ginseng supplementation may hold promise in alleviating the symptoms of CI in individuals infected with COVID‐19 [153].

#### 2.2.5 EGb 761

It is a flavonoid extract derived from the *Ginkgo biloba* tree. In a case series study, when administered at a dosage of 2 × 80 mg daily, it resulted in significant improvements in concentration and attention deficits (brain fog) in post COVID‐19 patients [154].

#### 2.2.6 Co‐ultraPEALut

It is a nutraceutical compound, a combination of luteolin (an antioxidant) and PEA (Palmitoylethanolamide) which acts as a neuroprotective and anti‐inflammatory agent. This study [155] demonstrated that co‐ultraPEALut treatment resulted in a significant reduction in CIs in post‐COVID‐19 patients.

### 2.3 Nonpharmacological Treatments

#### 2.3.1 Noninvasive Brain Stimulation

NIBS refers to a set of approaches for modifying brain electrical activity in specific cortical areas, including neuronal excitability, neural plasticity, and changes in connection patterns [156]. In recent decades, various NIBS approaches, including tACS, TMS, and transcranial direct current stimulation (tDCS), have been tried for the treatment of CI in various psychiatric and neurological diseases, as well as in healthy cognitive aging [157]. A series of 20 sessions using intermittent theta‐burst stimulation (iTBS) on the left dorsolateral prefrontal cortex (DLPFC) and the right lateral orbitofrontal cortex improved executive functions in a cohort of 23 individuals [158]. In a 30‐year‐old woman, accelerated theta‐burst TMS applied to the right DLPFC, followed by intermittent theta‐burst TMS on the left DLPFC, produced marked improvements in memory [159]. A combination of tDCS and online cognitive training was more promising, leading to improvements in processing speed, verbal learning, and memory in four patients [160].

#### 2.3.2 Rehabilitation

In a study conducted by Ruchi and colleagues, the significance of in‐patient rehabilitation for individuals infected with COVID‐19 was investigated, and the relationship between cognition and functional improvement was explored. Their findings revealed a substantial improvement in both cognition and functional ability during in‐patient rehabilitation, with clinically meaningful cognitive enhancements showing a strong association with improved function [119]. Given the reduced accessibility of conventional in‐person rehabilitation during the COVID‐19 pandemic, researchers began advocating for remote, home‐based rehabilitation strategies tailored to those experiencing CI. These home‐based strategies, recommended for postdischarge patients, encompass exercise regimens, direct care, and telerehabilitation [120].

COVID‐19 rehabilitation (CR) programs have demonstrated substantial improvements in clinical outcomes. A cohort study conducted by Daynes et al. revealed enhancements in exercise capacity, reductions in respiratory symptoms, alleviation of fatigue, and cognitive improvement among post‐COVID individuals [121]. Notably, physical exercise programs have been empirically effective in enhancing various cognitive domains, including attention, processing speed, executive function, and memory. However, applications of exercise in broader populations have shown an increase in exercise engagement without substantial improvements in functional abilities, such as motor skills and cognition [161]. Therefore, it is essential that rehabilitation tasks are customized to address the specific needs of individual patients while simultaneously fostering social interaction and psychological well‐being to prevent feelings of isolation [122]. In a study conducted by Molina et al., the effects of a neurorehabilitation program for patients experiencing post‐COVID neurological symptoms were examined. This comprehensive program encompassed respiratory rehabilitative exercises, physical therapy exercises, and neuropsychological rehabilitation (NPR), a holistic approach involving mood intervention, compensatory strategies, and cognitive therapy. Notably, the exercise group demonstrated enhanced performance on memory‐related tasks [123].

Another remote rehabilitation strategy is cognitive telerehabilitation (CTR), wherein patients perform an intensive exercise regimen at home under the virtual supervision of a clinician using mobile applications. The selection of exercises is tailored to address each patient's specific neurological deficits [120]. CTR has proven to be particularly advantageous for patients residing in remote areas, older individuals, and during pandemic‐related restrictions. Despite the advantages of home‐based rehabilitation, it is important to acknowledge that implementing such programs for individuals with CI may reduce their contact and engagement with the external environment, potentially impacting their psychological well‐being.

#### 2.3.3 Cognitive Rehabilitation Training

Cognitive rehabilitation training (CRT) presents a promising approach to address CI, particularly in the context of the ongoing pandemic. Innovative techniques such as brain–computer interface (BCI), virtual reality (VR), and artificial intelligence (AI) have emerged as valuable tools for CRT. For instance, BCI enables direct and objective monitoring of specific brain responses in cognitively impaired patients, providing accurate insights that can be applied both in community settings and within the patient's home. Conversely, VR‐based approaches create immersive environments for CI patients, encouraging greater engagement and enhancing the rehabilitation experience. The integration of VR technology into exercise rehabilitation holds the potential to enhance the psychological well‐being of CI patients [124]. Furthermore, the implementation of robot‐assisted training programs designed to target multiple cognitive domains over a 6‐week period among older patients with mild CI has yielded significant enhancements in their overall cognitive function [125]. Combining physical training with concurrent tDCS may further enhance and sustain the behavioral effects of the training regimen. Research involving anodal tDCS applied to the frontal brain regions during working memory practice has demonstrated beneficial effects on both trained and untrained memory functions, as well as daily life activities. Ongoing research by Thams et al. is extending this program to patients experiencing post‐COVID‐19 CI [126].

#### 2.3.4 Combined Therapy

As discussed earlier, the increase in physical activity through rehabilitation methods plays a pivotal role in ameliorating cognitive dysfunction. A recent meta‐analysis of randomized controlled trials has underscored the synergistic benefits of combining cognitive and physical rehabilitation. This combined approach yields more pronounced effects compared to either physical or cognitive rehabilitation alone, particularly with respect to neurological functions, encompassing memory, global cognition, executive functions, and attention. These benefits are particularly noteworthy among elderly individuals with CI [127].

## 3 Conclusion

The impact of COVID‐19 extends far beyond its acute viral phase, giving rise to a complex and challenging long COVID syndrome, including PCNS characterized by CI and various neurological symptoms. This presents a significant challenge to healthcare systems worldwide. The pathophysiology of CI in COVID‐19 patients is multifaceted, unveiling numerous avenues for targeted therapy. Emerging treatment modalities provide a glimmer of hope, encompassing anti‐inflammatory therapies, drugs targeting the RAS, NLRP3 inflammasome, RYR2 channels, viral entry pathways, nutraceuticals, and rehabilitation programs. Among these, anti‐inflammatory therapies and individualized cognitive rehabilitation programs hold significant promise for cognitive enhancement in affected individuals. However, it is important to note that most evidence regarding drug efficacy is derived from in vitro and in vivo studies, which may not directly translate to humans. These findings must be interpreted with caution, highlighting the critical need for further clinical research to validate and refine these therapeutic strategies. Incorporating the latest mechanistic insights, this review underscores the urgency of addressing COVID‐19‐related CI as a public health priority. Although numerous therapeutic avenues exist, most require rigorous clinical evaluation. By identifying key pathophysiological drivers and outlining promising therapeutic strategies, we provide a roadmap for future research and clinical interventions. Addressing post‐COVID cognitive dysfunction will require collaborative efforts from neuroscientists, clinicians, and public health experts to mitigate its long‐term impact on global health.

## Author Contributions

Y.‐H.C., J.‐S.J., S.‐Y.T., and C.‐Y.H. conceived the concept of the review paper. C.‐H.Y., T.‐L.Y., T.T.D.L., and M.K.S. drew and prepared the images and tables. Y.‐H.C., J.‐S.J., S.A., S.‐Y.T., and C.‐Y.H. wrote the manuscript. All the authors discussed, edited, and approved the final version of the manuscript.

## Conflicts of Interest

The authors declare no conflicts of interest.

## Data Availability

Data sharing not applicable to this article as no datasets were generated or analysed during the current study.
